# Mediating role of perceived stress on the association between domestic violence and postpartum depression: cross-sectional study in Bangladesh

**DOI:** 10.1192/bjo.2022.633

**Published:** 2023-01-18

**Authors:** Fowzia Tasnim, Sumaiya Abedin, Md. Mosfequr Rahman

**Affiliations:** Department of Population Science and Human Resource Development, University of Rajshahi, Rajshahi, Bangladesh

**Keywords:** Depressive disorders, domestic violence, perceived stress, postpartum depression, mediation

## Abstract

**Background:**

Postpartum depression (PPD) is a major depressive disorder developed after childbirth that negatively affects the well-being of both mother and infant. The relationship between domestic violence and the development of PPD symptoms is well documented. However, empirical evidence is lacking on how a person's perception of stress mediates this relationship.

**Aims:**

To estimate the degree to which perceived stress may explain the association between being the victim of domestic violence and developing PPD symptoms among Bangladeshi mothers.

**Method:**

A cross-sectional survey design was employed from October to December 2019 to collect data from 497 postpartum mothers within the first 6 months of giving birth. The associations between domestic violence victimisation and developing PPD symptoms were assessed using multivariable logistic regressions. The Karlson–Holm–Breen method was used for mediation analysis.

**Results:**

One-third (34%) of the mothers in this sample reported experiencing PPD within 6 months. A one-item increase in the number of reported experiences (‘items’) of controlling behaviour, emotional domestic violence and physical domestic violence increased the odds of developing PPD symptoms by 27%, 40% and 31% respectively, after controlling for other variables and mediators. Furthermore, after adjusting for other variables, the mediating effect of perceived stress on the association of controlling behaviour, emotional domestic violence, physical domestic violence and any form of domestic violence with developing PPD symptoms was 45.1%, 43.0%, 31.2% and 37.5% respectively.

**Conclusions:**

Findings suggest that perceived stress partially mediates the association between domestic violence victimisation and developing PPD symptoms. Understanding these complex relationships may help policymakers to formulate appropriate intervention strategies and support services.

Postpartum depression (PPD) is a non-psychotic mood or mental disorder and a very common health condition in women after childbirth.^1^ It typically manifests within 12 months postpartum,^2^ affecting an estimated 17.2% of reproductive-aged women globally (ranging from 6.5% in Denmark to 60.9% in Afghanistan).^3^ Untreated PPD has multiple short- and long-term negative effects on both mothers and children, including maternal suicide,^4^ poor infant and child development^5^ and depression in partners.^6^

Given its adversity, there is a great interest in identifying factors associated with PPD. Although pregnancy- and childbirth-related hormonal and other physiological changes are believed to be the main causes of PPD,^7^ various psychological, socioeconomic and cultural factors are also reported to be linked to the development of PPD among women.^8–10^ A recent umbrella review by Hutchens & Kearney^11^ documents that previous depressive episodes, antenatal depression, life stress and lack of social support have statistically significant relationships with developing PPD. A handful of studies documented that husband/partner-perpetrated violence increases the likelihood of PPD among mothers.^12–16^

Domestic violence is a common global phenomenon causing increasing public health concern.^17^ Although in most cases the husband or intimate partner is the perpetrator, reports of domestic violence against women by the family of their husband or partner are also available.^16,18^ The association between domestic violence in the form of intimate partner violence and PPD has been reported;^8,12,19^ however, their causal links are still not well documented. There is a less clear understanding of whether experience of domestic violence by the mother and subsequent development of PPD are linked through other individual factors or adversities, such as maternal perceived stress. Moreover, the stress process theory^20^ is not yet introduced in existing research to explain the relationship between victimisation with subsequent mental health outcomes.

The stress process could help explain the link between victimisation and subsequent mental health outcomes through its conceptual domains. This model explains in detail the relationship between primary sources of stress (e.g. adverse life events) and the manifestation of stress (e.g. negative mental health outcomes) and acknowledges other underlying individual and social processes affecting the main relationship.^20^ In applying these conceptual domains of the stress process in the current study, we would expect that a woman's experience of domestic violence (i.e. the sources of stress) might not be directly related to the development of PPD (i.e. the manifestation of stress); rather, this relationship would be indirectly linked through other individual adversities, such as perceived stress (i.e. the mediators of stress). Examining individual adversities that might affect the relationship between the source of stress (i.e. experiencing domestic violence) and the manifestation of stress (i.e. developing PPD) enhances the understanding as to whether experiencing negative life events and the subsequent negative health outcomes might follow a more complex pattern.^20^ Therefore, this study aimed to analyse the link between lifetime domestic violence from husbands and/or in-laws and the development of PPD among recent mothers in Bangladesh and to estimate the degree to which perceived stress might explain the relationship (Fig. 1).

**Fig. 1:**
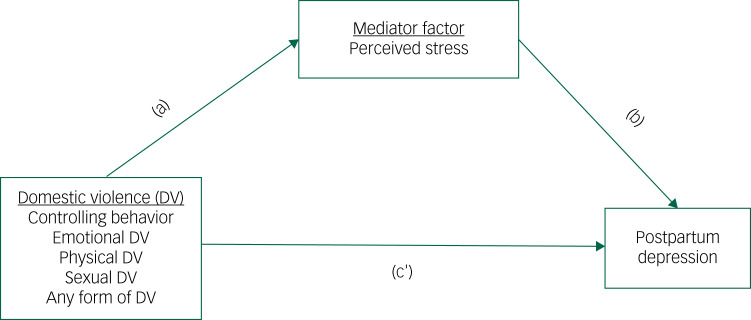
Hypothesised framework of the mediation analysis. c', direct effect of domestic violence on postpartum depression controlling for perceived stress.

## Method

### Participants and procedure

**Fig. 2:**
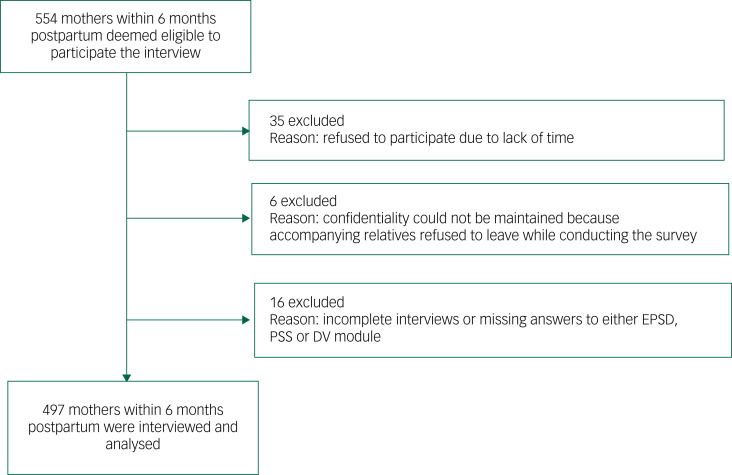
Participant flow diagram. EPDS, Edinburgh Postnatal Depression Scale; PSS, Perceived Stress Scale.

This study is based on a cross-sectional survey that was conducted in an urban primary health centre in Rajshahi, a major divisional city corporation in Bangladesh, from October to December 2019. Respondents attended the health centre for their children's vaccination. The eligibility criterion for participation in the study was that the mothers had to be within 6 months postpartum. The sample size was calculated using a single population proportion formula for cross-sectional studies with the following assumptions: the 6-month PPD prevalence in Bangladesh was 35%,^15^ the admissible error was 5% and a 95% confidence level. The theoretical sample size was calculated to be 466 mothers, which included an extra 10% to allow for non-response during the study, a design effect (1.2) and an adjustment for a finite population. In practice, we were able to invite 554 eligible postpartum mothers to participate in the study. Nearly 90% (*n* = 497/554) gave their written consent to participate and completed the interview voluntarily (Fig. 2). Sociodemographic, mental health, stress and domestic violence-related data from these 497 women were collected. The data collection procedure is described elsewhere.^16^ Written informed consent was obtained from all respondents. The authors assert that all procedures contributing to this work comply with the ethical standards of the relevant national and institutional committees on human experimentation and with the Helsinki Declaration of 1975, as revised in 2008. All procedures involving human participants/patients were approved by the Technical Review Committee, comprising members from the Health Division of Rajshahi City Corporation and the Urban Primary Health Care Project (A-209(07)/126/RCC).

### Variables and measures

The outcome variable, maternal PPD, was evaluated using the Edinburgh Postnatal Depression Scale (EPSD). The EPSD is a self-reported 10-item instrument that is easily administrable and a commonly used method for screening for PPD.^21^ Each item of the EPSD is scored from 0 to 3, and the sum of 10 items makes the total maximum score 30. According to a prior study conducted in Bangladesh, the Bangla version of the EPSD has a sensitivity of 89% and a specificity of 87% at the optimum cut-off score of 10.^22^ Therefore, we used a threshold of 10 to define PPD among mothers. The reliability of the EPSD was 0.82 according to Cronbach's α in the present study.

Experience of lifetime domestic violence was the main exposure of interest. A shortened modified version of the Conflict Tactics Scale^23^ was used to assess experience of domestic violence among these mothers. This retrospective self-reported scale includes behaviourally specific questions, which encourages the greater disclosure of experiencing violence than asking respondents to identify themselves as abused or battered.^24^ Based on qualitative formative research, the questionnaire was adapted for use in Bangladesh and revised to reflect domestic violence perpetrated by an intimate partner and/or a family member.^25^ The respondents were asked whether their husbands or in-laws had carried out specific acts of physical, sexual or emotional abuse or acted in a controlling way. Physical violence was measured in this study as the experience of any physically aggressive behaviours that potentially cause harm, injury or even death, such as slapping, hitting, kicking and beating. Experience of forced sexual intercourse or other forms of sexual coercion was used to assess sexual violence. Emotional violence was defined as the experience of any behaviours that cause emotional harm or trauma, such as insults, belittling, constant humiliation and intimidation. Controlling behaviour included behaviours that are imposed on someone to control him/her (e.g. movement monitoring; isolating from family and friends). The questions used to assess domestic violence are available elsewhere.^16^

We hypothesised that perceived stress partly explains the associations of PPD with different forms of domestic violence. The Perceived Stress Scale (PSS) was used to measure women's perceived stress. The PSS is a two-factor ten-item self-report tool typically used to evaluate an individual's perception of stressful life events.^26^ Summation of all the items produces a total score that ranges from 0 to 40, where higher scores indicate increased stress. The internal reliability of the PSS in this sample was adequate (Cronbach's α = 0.77).

Finally, our study included several control variables that have been theoretically and empirically associated with the development of PPD.^27–33^ These are: maternal age (categorised as 15–24, 25–34 or 35–49 years), age at marriage (<18 or ≥18 years), educational attainment (primary, secondary or higher), parity (1, 2 or ≥3), pregnancy intention (intended or unintended), experiencing any complication during pregnancy or childbirth (yes or no), pregnancy duration (<37 weeks or ≥37 weeks), and newborn's birth weight (<2500 g or ≥2500 g). We also included the ‘wealth index’ to assess the women's socioeconomic position, which is created using household assets, including durable goods (e.g. freezer, television, radio), transport vehicles (car, motorcycle, bicycle), and whether the household has electricity.^34^ Principal component analysis (PCA) was used to develop the wealth index. A normalised index is produced using PCA which has a mean value of zero and a standard deviation (s.d.) of one. A score for each asset was assigned to each household, and the scores were summed by household. Women were assigned according to the total score of the household in which they lived. The wealth index categorised women into tertiles: from one (poor) to three (rich).

### Statistical analysis

We conducted *t*-tests and chi-square analyses, where appropriate, to determine differences in either means or proportions among all analytic variables based on the presence of the symptoms of PPD. We examined the correlations among outcomes, exposures and potential mediators using Pearson's correlation. In total, we tested ten hypotheses. The problem of multiple hypotheses testing was accounted for by setting the threshold for significance at *P* = 0.05/10 = 0.005 using Bonferroni correction. Using multivariable logistic regression models, we assessed the relationships between the development of PPD and different forms of domestic violence experienced by mothers in their lifetime, controlling for other sociodemographic and health-related variables. Since a high correlation existed among the independent variables, i.e. physical domestic violence, sexual domestic violence, controlling behaviour, emotional domestic violence and any form of domestic violence, we used separate models to examine the relationships. Two models were constructed for each of the independent variables: in the first model, the potential mediator was excluded and adjusted for other controlling variables; in the second model the potential mediator was included in addition to other variables. Using these models, we calculated odds ratios (ORs) and the 95% confidence intervals.

The mediation analysis was conducted using a logistic regression based on the Karlson–Holm–Breen (KHB) method.^35^ In this method, the total effect (i.e. unadjusted for the mediator) is deconstructed into direct effect (i.e. the effect of domestic violence on PPD, controlling for the mediator) and indirect effect (i.e. the mediating effect). We calculated the percentage of the association between domestic violence experience and PPD explained by stress (mediated percentage), which was done by dividing the indirect effect by the total effect. All statistical analyses were done using Stata 16.0 for Windows.

## Results

Nearly half of respondents (42.4%) belonged to the age group 15–24 years; 39.2% of women reported that they were married before the age of 18; and 45.7% of women were from the poor wealth index category (Table 1). Approximately one-quarter (24.5%) of the most recent births were reported to be unintended by their mothers, and 18.5% of mothers reported experiencing complications during pregnancy/childbirth. The prevalence of PPD within 6 months of childbirth reported in this sample was 34%.

**Table 1:** Descriptive statistics for the study variables

Variables	Total sample (*n* = 497)	Postpartum depression	
		Yes	No
Age group, years: *n* (%)			
15–24	211 (42.4)	67 (31.7)	144 (68.3)
25–34	246 (49.5)	90 (36.6)	156 (63.4)
35–49	40 (8.1)	12 (30.0)	28 (70.0)
Age at marriage, *n* (%)			
<18 years	195 (39.2)	80 (41.0)	115 (59.0)
≥18 years	302 (60.8)	89 (29.5)	213 (70.5)
Education, *n* (%)			
Primary	154 (31.0)	47 (30.5)	107 (69.5)
Secondary	128 (25.7)	52 (40.6)	76 (59.3)
Higher	215 (43.3)	70 (32.6)	145 (67.4)
Wealth index, *n* (%)			
Poor	227 (45.7)	78 (34.4)	149 (65.6)
Middle	130 (26.2)	35 (26.9)	95 (73.1)
Rich	140 (28.2)	56 (40.0)	84 (60.0)
Parity, *n* (%)			
1	241 (48.5)	82 (34.0)	159 (66.0)
2	190 (38.2)	60 (31.6)	130 (68.4)
≥3	66 (13.3)	27 (40.9)	39 (59.1)
Pregnancy intention, *n* (%)			
Intended	375 (75.5)	102 (27.2)	273 (72.8)
Unintended	122 (24.5)	67 (54.9)	55 (45.1)
Complications during pregnancy/childbirth, *n* (%)			
No	405 (81.5)	125 (30.9)	280 (69.1)
Yes	92 (18.5)	44 (47.8)	48 (52.2)
Duration of pregnancy, *n* (%)			
<37 weeks	300 (60.4)	111 (37.0)	189 (63.0)
≥37 weeks	197 (39.6)	58 (29.4)	139 (70.6)
Birth weight, *n* (%)			
<2500 g	41 (8.3)	16 (39.0)	25 (61.0)
≥2500 g	456 (91.7)	153 (33.5)	303 (66.5)
Sexual domestic violence, yes: *n* (%)	88 (17.7)	37 (42.1)	51 (57.9)
Controlling behaviour, no. of CTS items (0–4): mean (s.d.)	0.59 (0.96)	0.90 (1.21)	0.43 (0.76)
Emotional domestic violence, no. of items (0–4): mean (s.d.)	0.79 (1.07)	1.24 (1.24)	0.56 (0.90)
Physical domestic violence, no. of CTS items (0–6): mean (s.d.)	0.71 (1.29)	1.09 (1.51)	0.51 (1.12)
Any forms of domestic violence, no. of CTS forms (0–4): mean (s.d.)	1.23 (1.30)	1.69 (1.34)	0.99 (1.23)
PSS score (range: 0–40): mean (s.d.)	21.45 (5.02)	24.34 (4.88)	20.00 (4.44)

CTS, Conflict Tactics Scale; PSS, Perceived Stress Scale.

The mean PSS score for perceived stress reported by mothers in this sample was 21.45 (s.d. = 5.02) out of a possible range of 0–40. On the Conflict Tactics Scale, the average number of forms of controlling behaviour (‘items’) experienced by mothers was 0.59 out of a possible range of 0–4, while the average number of emotional domestic violence items was 0.79 out of a possible range of 0–4. The mean number of physical domestic violence items reported by mothers was 0.71 out of a possible range of 0–6; the mean number of different forms of domestic violence (controlling behaviour, emotional violence, physical violence or sexual violence) experienced by mothers was 1.23 out of a possible range of 0–4 (Table 1).

The mean PSS score was higher among mothers who developed PPD symptoms than those who did not (mean 24.34 (s.d. = 4.88) *v*. 20.00 (s.d. = 4.44)). Similarly, the score for experiencing any form of domestic violence among mothers who developed PPD symptoms was higher than among mothers who did not (mean 1.69 (s.d. = 1.34) *v*. 0.99 (s.d. = 1.23)) (Table 1).

The correlation matrix in Table 2 presents correlations between PPD, controlling behaviour, emotional domestic violence, physical domestic violence, sexual domestic violence, any form of domestic violence and perceived stress. A higher number of experiences of controlling behaviour (*r* = 0.221), emotional domestic violence (*r* = 0.296), physical domestic violence (*r* = 0.147) and any form of domestic violence (*r* = 0.210) was correlated with higher levels of perceived stress.

**Table 2:** Correlation between outcome, exposures and potential mediators

Variables	1	2	3	4	5	6	7
Postpartum depression	1.000						
Controlling behaviour	0.234***	1.000					
Emotional domestic violence	0.297***	0.543***	1.000				
Physical domestic violence	0.212***	0.386***	0.481***	1.000			
Sexual domestic violence	0.079	0.273***	0.257***	0.346***	1.000		
Any form of domestic violence	0.251***	0.674***	0.721***	0.685***	0.658***	1.000	
Perceived stress	0.407***	0.221***	0.296***	0.147**	0.011	0.210***	1.000

***P < 0.001, **P < 0.01.

Results from the logistic regression analysis are displayed in Table 3. Each form of domestic violence except sexual domestic violence was associated with higher odds of developing PPD symptoms, after adjusting for other variables without perceived stress. When perceived stress was added to the model, each form of domestic violence remained associated with PPD but to a lesser extent. After controlling for all other variables and potential mediators, a one-item increase in the number of reported experiences (‘items’) of controlling behaviour, emotional domestic violence and physical domestic violence on the Conflict Tactics Scale increased the odds of developing PPD symptoms by 27%, 40% and 31% respectively. Perceived stress was strongly associated with developing PPD symptoms in all models, as every one-point increase in the PSS score was significantly associated with developing PPD symptoms with higher odds.

**Table 3:** Logistic regression analysis of the association of different forms of domestic violence and perceived stress with postpartum depression

Variables	Postpartum depression									
	OR (95% CI)^a^	OR (95% CI)^b^	OR (95% CI)^a^	OR (95% CI)^b^	OR (95% CI)^a^	OR (95% CI)^b^	OR (95% CI)^a^	OR (95% CI)^b^	OR (95% CI)^a^	OR (95% CI)^b^
Controlling behaviour	1.51 (1.20–1.88)^c^	1.27 (1.02–1.63)								
Emotional domestic violence			1.72 (1.41–2.10)^c^	1.40 (1.13–1.74)^c^						
Physical domestic violence					1.39 (1.18–1.63)^c^	1.31 (1.09–1.56)^c^				
Sexual domestic violence							1.38 (0.83–2.31)	1.50 (0.85–2.66)		
Any form of domestic violence									1.44 (1.23–1.70)^c^	1.29 (1.08–1.54)^c^
Perceived stress		1.21 (1.14–1.27)^c^		1.25 (1.14–1.27)^c^		1.32 (1.19–2.42)^c^		1.20 (1.12–1.39)^c^		1.27 (1.14–1.35)^c^

a. Model excludes perceived stress (potential mediator) and was adjusted for age, age at marriage, education, wealth index, unintended pregnancy, parity, complications during pregnancy/childbirth, duration of pregnancy and birth weight.

b. Model includes perceived stress (potential mediator) and was also adjusted for age, age at marriage, education, wealth index, unintended pregnancy, parity, complications during pregnancy/childbirth, duration of pregnancy and birth weight.

c. Using Bonferroni correction for multiple tests the adjusted *P*-value is 0.005 for significance.

Table 4 reports the adjusted results of the mediation analysis conducted using the KHB method. The total effect of controlling behaviour on developing PPD symptoms was an odds ratio of 1.78 (95% CI 1.40–2.27). There remained a direct effect of controlling behaviour on developing PPD symptoms independent of perceived stress (potential mediator) (OR = 1.37, 95% CI 1.08–1.74). The odds ratio for the indirect effect of controlling behaviour on developing PPD symptoms through perceived stress was 1.30 (95% CI 1.16–1.45), indicating that there was a mediated effect with perceived stress, which accounts for 45.1% of the total effect of controlling behaviour on developing PPD symptoms, accounting for all other covariates. For other domestic violence exposures, after adjusting for control variables, the mediating effect of perceived stress on the association of emotional, physical and any form of domestic violence with developing PPD symptoms was 43.0%, 31.2% and 37.5% respectively. Since the indirect effect of sexual domestic violence on developing PPD symptoms through perceived stress was not significant, we did not calculate the mediated percentage for this association.

**Table 4:** The adjusted results of mediation of stress on the association between different forms of domestic violence and postpartum depression (Karlson–Holm–Breen method)^a^

Mediator	Independent variable: controlling behaviour			Independent variable: emotional domestic violence			Independent variable: physical domestic violence			Independent variable: sexual domestic violence			Any form of domestic violence		
	OR 95% CI	*P*^b^	% Mediated	OR 95% CI	*P*^b^	% Mediated	OR 95% CI	*P*^b^	% Mediated	OR 95% CI	*P*^b^	% Mediated	OR 95% CI	*P*^b^	% Mediated
Stress															
Total	1.78 (1.40–2.27)	<0.001		1.92 (1.55–2.38)	<0.001		1.47 (1.23–1.75)	<0.001		1.47 (0.88–2.45)	0.125		1.56 (1.31–1.85)	<0.001	
Direct	1.37 (1.08–1.74)	0.003		1.45 (1.18–1.79)	<0.001		1.30 (1.10–1.54)	0.003		1.43 (0.86–2.38)	0.139		1.32 (1.11–1.56)	<0.001	
Indirect	1.30 (1.16–1.45)	<0.001	45.1	1.32 (1.19–1.48)	<0.001	43.0	1.13 (1.04–1.21)	0.002	31.2	1.03 (0.88–1.21)	0.898		1.18 (1.09–1.28)		37.5

a. Models were adjusted for age, age at marriage, education, wealth index, parity, unintended pregnancy, complications during pregnancy/childbirth, duration of pregnancy and birth weight. The mediated percentage was calculated only when the indirect effect was significant.

b. Using Bonferroni correction for multiple tests the adjusted *P*-value is 0.005 for significance.

## Discussion

This study assesses the relationship between exposure to different forms of domestic violence and the risk of developing PPD among mothers within 6 months postpartum as well as determining whether perceived stress mediates these relationships. Specifically, we tested two hypotheses: (a) that all forms of domestic violence would predict the development of PPD symptoms; and (b) that perceived stress would mediate the relationship between different forms of domestic violence and the development of PPD symptoms. This study found that 34% of the postpartum mothers reported developing PPD symptoms within 6 months of delivery, which is consistent with findings of earlier studies conducted in Bangladesh.^8,15,36^ In support of our first hypothesis, the findings suggest that the experience of an increasing number of items of controlling behaviour, emotional domestic violence and physical domestic violence was associated with higher odds of developing PPD symptoms. Our findings extend support to existing research which has found that experiencing violence from a husband/partner predicts adverse mental health in women, especially during the postpartum period.^8,12–15,36^

Although the findings of this study are similar to the findings of past studies, comparability is limited as previous studies only consider violence perpetrated by husbands/partners, ignoring the violence perpetrated by in-laws. This study included both husband- and/or in-law-perpetrated domestic violence, and therefore these findings might be of particular interest to other researchers and policymakers. Furthermore, since in South Asian countries, after marriage women generally reside in their husband's parents’ home, sharing the home with their in-laws, specifically their mothers-in-law, the findings of this study could be helpful in identifying PPD risk factors and addressing them. Additionally, it is already reported in many studies from low- and middle-income countries that women not only experience violence from their male partner but also a significant proportion of women (ranging from 5% to 36%) experience violence from members of their partner's family (in-laws).^37,38^

The findings of this study also support our second hypothesis, in which we anticipated that other individual adversities, such as perceived stress, would indirectly link (i.e. mediate) the experience of victimisation and its subsequent mental health repercussions. Our findings suggest the existence of mediating effects of perceived stress in the relationship between experiencing different forms of domestic violence and the development of PPD symptoms. These findings are consistent with the study of Stoliker,^39^ where the author reported that perceived stress has a mediating effect in the relationship between some forms of victimisation (e.g. personal victimisation, household/property victimisation, cyberbullying and physical/sexual ex-partner abuse) and psychological health outcomes, such as self-reported mental health and life satisfaction. It is well documented that being the victim of domestic violence is related to increased experiences of stress^40,41^ and stress-related mental health problems such as depression.^16,42^ Higher perceived stress is negatively associated with self-efficacy,^43^ coping resources and associated behaviours,^44^ all of which influence future vulnerability. Additionally, according to the stress-generation hypothesis, the experience of an increased number of stressors or negative events predicts future sensitivity to stress and ill health.^45^ The current study suggests that domestic violence victimisation elicits stress, which leads to the development of PPD symptoms.

Earlier studies documented that the prevalence of stress and PPD was higher among mothers who experienced sexual domestic violence than mothers who did not;^40,46^ however, corroborating a previous study,^40^ the mediating effect of stress in the association of sexual domestic violence with PPD symptoms was not identified in this study. In fact, this study found no significant relationship between sexual domestic violence victimisation and PPD. This could be due to participants’ underreporting of the experience of husband- or in-law-perpetrated sexual violence. In the South Asian context, married women are generally unwilling to talk about sex or sexual acts.^47^

### Limitations

Limitations of this study should be considered when interpreting the results. We are unable to make causal inferences on associations of interest because of the cross-sectional nature of the study. For example, there could be a bidirectional relationship between domestic violence victimisation, perceived stress and developing PPD symptoms, i.e. the development of PPD symptoms might predict domestic violence victimisation. Future longitudinal research could help to determine the temporal ordering of variables and ascertain the cause-and-effect relationships. However, the evidence suggests that the experience of domestic violence adversely affected the psychological health of postpartum mothers, which could also be linked through the perception of stress. We used a retrospective self-report instrument (the Conflict Tactics Scale) to measure domestic violence; therefore, there could be substantial recall bias as well as an increased likelihood of underreporting. Our measures of developing PPD symptoms and perceived stress (the EPSD and PSS-10) are widely used screening instruments but they are not diagnostic tools, therefore they might not reflect the actual prevalence of these mental health problems. In addition, since the risk period for developing PPD symptoms is documented up to 12 months postpartum,^2^ the prevalence of PPD reported in this study should be interpreted with caution as it only represents the PPD among women within 6 months of delivery. This study was based on a sample collected from an urban setting; therefore, findings cannot be generalised to rural women or all women in Bangladesh. Owing to budget constraints and time limitations, we restricted maternal domestic violence to only being husband- and/or in-law-perpetrated and were unable to investigate broader experiences of domestic violence. Moreover, using a joint question in assessing domestic violence perpetrated by either husbands or in-laws limits us in separately assessing the effects of domestic violence perpetrated by in-laws. Finally, for this same reason, we were not able to collect data on some important variables, such as antepartum depression or previous episodes of depression and women's social support, personality and coping strategies, which might have significant association with stress and the risks for developing PPD.

### Implications and future research

Despite these limitations, the study's findings have important implications for policymakers and clinicians. The high prevalence of PPD and its relationship with domestic violence and the potential mediator, perceived stress, can primarily provide an important understanding of the relationships and help promote appropriate interventions to improve women's social and mental health. The results also underscore the importance of healthcare professionals’ understanding of the relationship between domestic violence and PPD as a basis for providing appropriate clinical care for Bangladeshi women attending healthcare facilities. Assessing the experience of domestic violence, identifying and treating stress, and providing women with required social support can be important strategies that might reduce the development of PPD symptoms among domestic violence survivors. Additionally, routine screening for maternal depression in antenatal and postnatal care should be introduced. This can be achieved if health staff are appropriately trained to screen for depression as a routine component of antenatal and postnatal care. Awareness of the negative consequences of domestic violence, stress and PPD on both mother and child needs to be increased. Mass media can play an important role in this regard. Finally, an empowerment approach, as suggested in Garcia et al's study,^40^ to reduce the likelihood that abused women will experience stress and their subsequent risk for developing depression might be utilised in future interventions among abused Bangladeshi women. Furthermore, policymakers from other male-dominant societies similar to Bangladesh, where the prevalence of domestic violence and mental health problems is high but not a focus for services, can use these findings in formulating their intervention strategies.

This study confirms the complex interrelationships between domestic violence victimisation, stress and PPD. Future studies could explore how other individual and social factors^48^ might affect the relationship and elucidate the processes resulting from domestic violence victimisation and the development of PPD symptoms.

## Data Availability

Data are available from the corresponding author on request.
